# Transcriptomic Insights into Higher Anthocyanin Accumulation in ‘Summer Black’ Table Grapes in Winter Crop Under Double-Cropping Viticulture System

**DOI:** 10.3390/plants14010026

**Published:** 2024-12-25

**Authors:** Congqiao Wang, Chengyue Li, Youhuan Li, Yue Zeng, Jie Jiang, Linhui Wu, Siyu Yang, Dan Yuan, Lifang Chen, Zekang Pei, Viola Kayima, Haidi Liu, Zhipeng Qiu, Dongliang Qiu

**Affiliations:** 1 Key Laboratory of Ministry of Education for Genetics, Breeding and Multiple Utilization of Crops, College of Horticulture, Fujian Agriculture and Forestry University, Fuzhou 350002, China; wcq1333983179@163.com (C.W.); 18999752899@163.com (C.L.); lyl1233334@163.com (Y.L.); 13667961391@163.com (Y.Z.); 18859115232@163.com (J.J.); 18259908335@163.com (L.W.); 15163825210@163.com (S.Y.); 5220330060@fafu.edu.cn (D.Y.); 13509319672@163.com (L.C.); 13395088271@163.com (Z.P.); violakayima@gmail.com (V.K.); 18354630950@163.com (H.L.); 2 Lunong Agricultural Technology Co., Ltd., Xiamen 361100, China

**Keywords:** *Vitis vinifera* L., anthocyanin, double cropping, *VvOMT*, *VvMYB*, *VvbHLH*, *VvHY5*, *VvUFGT*

## Abstract

Anthocyanins are responsible for grape (*Vitis vinifera* L.) skin color. To obtain a more detailed understanding of the anthocyanin regulatory networks across’ the summer and winter seasons in grapes under a double-cropping viticulture system, the transcriptomes of ‘Summer Black’ grapes were analyzed using RNA sequencing. The average daily temperature during the harvest stage in the summer crop, ranging from 26.18 °C to 32.98 °C, was higher than that in the winter crop, ranging from 11.03 °C to 23.90 °C. Grapes from the winter crop accumulated a greater content of anthocyanins than those from the summer crop, peaking in the harvest stage (E-L38) with 207.51 mg·100 g^−1^. Among them, malvidin-3-O-glucoside (Mv-3-G) had the highest monomer content, accounting for 32%. The content of Cy-3-G during winter increased by 55% compared to summer. KEGG analysis indicated that the flavonoid biosynthesis and circadian rhythm—plant pathways are involved in the regulation of anthocyanin biosynthesis during fruit development. Pearson’s coefficient showed significant positive correlations between anthocyanin content and the *VvDFR*, *VvUFGT*, *VvOMT*, *VvMYB*, and *VvbHLH* genes in the winter crop; at full veraison stage, their expressions were 1.34, 1.98, 1.28, 1.17, and 1.34 times greater than in summer, respectively. The higher expression of *VvUFGT* and *VvOMT* led to higher contents of Cy-3-G and Mv-3-G in the winter berries, respectively.

## 1. Introduction

Grapevines (*Vitis vinifera* L.) are a very important and economically significant fruit crop cultivated on a large scale worldwide. China ranks as the second-largest country in terms of vineyard area and is the foremost producer of table grapes, representing 34% of the global total production. The color of grapes’ skin significantly influences the market value of table grapes. Anthocyanins are responsible for the vibrant red, violet, and blue colors of grapes [1,2]. Anthocyanins perform a multitude of biological roles, including protecting against damage caused by oxidative stress [3], defending against pathogenic assaults [4], neutralizing free radicals, and safeguarding against cardiovascular diseases and cancer [5,6]. These functions contribute to a myriad of health benefits. Consequently, there is an increasing focus on improving anthocyanin production in grapes during their growth stages, driven by the desire to promote health benefits and economic incentives [7,8].

Grape varieties produce anthocyanins via the flavonoid pathway. Anthocyanin biosynthesis can be divided into four steps. In the first stage, the direct precursor of anthocyanins, phenylalanine, is catalyzed to produce 4-coumaroyl-CoA. In the second stage, 4-coumaroyl-CoA is catalyzed to generate dihydroquercetin and dihydromyricetin [9,10]. In the third stage, the synthesis of dihydrokaempferol, dihydroquercetin, and dihydromyricetin catalyzes the conversion to colored anthocyanin glycosides [11]. In the fourth step, anthocyanin glycosides can be catalyzed further by flavonoid 3-O-glucosyltransferase (UFGT) [12], o-methyltransferase (OMT) [13], and acyltransferase (AT), which are all kinds of stable anthocyanin glycosides formed by transferase modification [14]. Dp-3-G is catalyzed by VvOMT to form Pt-3-G and Mv-3-G, and then Pt-3-G is further catalyzed by VvOMT to form Mv-3-G. The precise mechanisms behind the transportation of anthocyanins are not completely clear; one prevailing notion is that the glutathione S-transferase (GST) protein family has a vital function in the transportation and retention of these substances within the vacuole [15,16].

Anthocyanin accumulation in grapes is influenced by several elements, such as environmental conditions and agronomic techniques in viticulture [7,16,17]. Several factors, such as vine vigor [18], sunlight exposure [19], and water deficit [20], have been found to influence anthocyanin modification in grapes. Several canopy management techniques have been used in vineyards to improve grape quality. Basal leaf removal and berry thinning are important agronomic practices in vineyards. Basal leaf removal is an important method of managing the vineyard canopy. Basal leaf removal refers to the deliberate removal of specific leaves surrounding the berry cluster. This approach aims to optimize the canopy of the grapevine and thus improve the microclimate by ensuring that the berry clusters receive sufficient sunlight. Leaf removal can be carried out after berry formation but before veraison [21]. Thinning is an agricultural technique that involves the removal of living parts of the grape to concentrate its resources and prevent cluster compactness [22]. Berry thinning is the process of cutting berries from clusters, which can be performed using scissors [21,23] or by removing the top of the clusters to form blunt clusters [24]. Research indicates that both leaf removal and berry thinning can increase the concentration of total soluble solids (TSS), titratable acids, and phenolic compounds, such as anthocyanins, in grapes [24,25].

Traditionally, grapes are typically a seasonal crop, primarily harvested annually in the summer. However, in several locations, a two-crop-a-year or double-crop strategy is employed to maximize productivity. The double-cropping viticultural system represents a distinct approach, involving pruning and chemical applications after the typical summer harvest, facilitating budding and subsequent grape collection even during the winter season [26,27,28]. Over the past decade, growers in subtropical southern China have increasingly embraced this viticultural system.

Currently, there remains an incomplete comprehension of the molecular processes involved in anthocyanin biosynthesis in grapes from a double-cropping model. Therefore, we set out to investigate the molecular processes and cultural practices that aid the accumulation of anthocyanins in grapes. The experiment was carried out at Lunong Agricultural Technology Co., Ltd., in Xiamen, Fujian, China, using a two-crop system each year to investigate the effect of the growing season (summer and winter) on the accumulation of anthocyanins in grapes and the expression of related genes during the period from the green berry stage to the harvest stage (E-L 33 to 38). Moreover, leaf removal and berry thinning treatments were applied to investigate the effects of cultural practices on anthocyanin production.

## 2. Results

### 2.1. Temperature Variations in Double-Cropping Cultivation of Grapes

**Figure 1:**
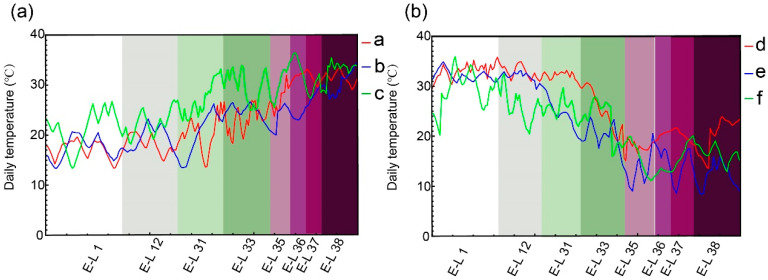
The mean daily temperature throughout the growth of grape berries. (**a**) Summer crop daily average temperature trends. (**b**) Winter crop daily average temperature trends. Developmental stages: pruning (E-L 1); 5 leaves separated (E-L 12); berry pea size (E-L 31); green berries (E-L 33); beginning of veraison (E-L 35); full veraison (E-L 36); end of veraison (E-L 37); and harvest stage (E-L 38). Points a, b, and c denote measurements at the front, middle, and back of the greenhouses for the summer crop. Points d, e, and f denote measurements at the front, middle, and back of the greenhouses for the winter crop.

There were seasonal temperature differences during the development period of the grapes under the double-cropping cultivation mode. By monitoring the temperature at the front, middle, and back of the experimental greenhouses, the average daily temperature from the pruning (E-L 1) to the harvest stage (E-L 38) showed an overall increasing trend from January to June, with temperatures during the harvest stage ranging from 26.18 °C to 32.98 °C (Figure 1a, Table S2). Conversely, the average daily temperature from the pruning (E-L 1) to the harvest stage (E-L 38) showed a decreasing trend from July to December, with temperatures during the harvest stage ranging from 11.03 °C to 23.90 °C (Figure 1b, Table S2).

### 2.2. Higher Anthocyanin Concentration in Winter Berries Compared to Summer Berries

**Figure 2:**
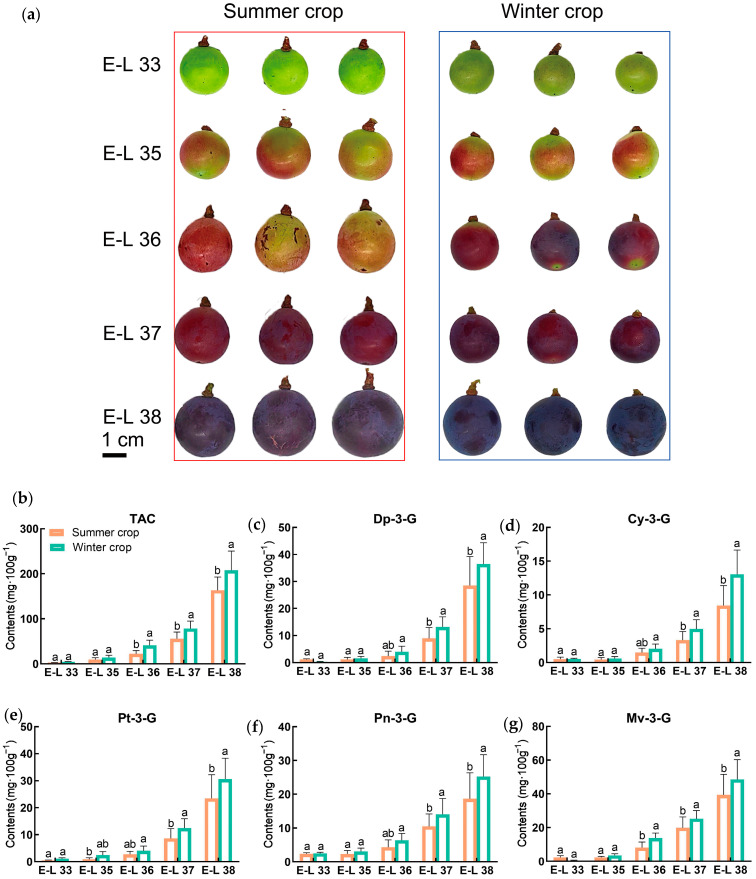
The effect of seasonal treatment on the content of anthocyanins in ‘Summer Black’ grape berries. (**a**) Grape berries in different developmental stages in winter and summer crops. (**b**) Total anthocyanin content in winter and summer crops. (**c**) Del-phinidin-3-O-glucoside (Dp-3-G) content in winter and summer crops. (**d**) Cyanidin-3-O-glucoside (Cy-3-G) content in winter and summer crops. (**e**) Petunidin-3-O-glucoside (Pt-3-G) content in winter and summer crops. (**f**) Peonidin-3-O-glucoside (Pn-3-G) content in winter and summer crops. (**g**) Malvidin-3-O-glucoside (Mv-3-G) content in winter and summer crops. Developmental stages: green berries (E-L 33); beginning of veraison (E-L 35); full veraison (E-L 36); end of veraison (E-L 37); and harvest stage (E-L 38). The vertical bars indicate the standard error of the mean (SEM). Statistical significance is denoted by distinct letters (*p* < 0.05).

Data analysis revealed significant differences between the winter and summer crop treated vines. Significant differences in size and color were observed between the summer and winter crop berries (Figure 2a). The winter crop berries showed elevated levels of total soluble solids (TSS) and pH compared to the summer berries. The summer crops had larger vine yields, individual berry weights, berry diameters, and berry lengths than the winter crops (Table S3).

The concentrations of both total and individual anthocyanins in berries from the winter crop were consistently higher as compared to the summer crop. The highest anthocyanin content was recorded in the E-L38 stage of the winter berries (Figure 2b), with 207.51 mg·100 g^−1^, which was 1.27 times the amount in the summer berries. The winter berries showed higher levels of Cy-3-G, Pn-3-G, Pt-3-G, Dp-3-G, and Mv-3-G, with increases of 55%, 35%, 31%, 28%, and 23%, respectively, during the harvest stage as compared to the summer berries (Figure 2c–g). Among them, Mv-3-G had the highest monomer content, accounting for 32%. The difference between the winter and summer crops was the greatest in the E-L 36 stage, where the Mv-3-G content in berries from the winter crop was 1.67 times greater than that of the summer crop. The sum of the five monomers was 154.01 mg·100 g^−1^ in the winter berries and 118.48 mg·100 g^−1^ in the summer berries. In both the summer and winter crops, the total anthocyanin content (TAC) and individual anthocyanin levels significantly increased from full veraison to harvest. After the E-36 stage, total and individual anthocyanin levels showed significant increases.

### 2.3. Evaluation of Transcriptome Sequencing Quality and Alignment Results for Double-Cropping Cultivated Grape Samples

**Figure 3:**
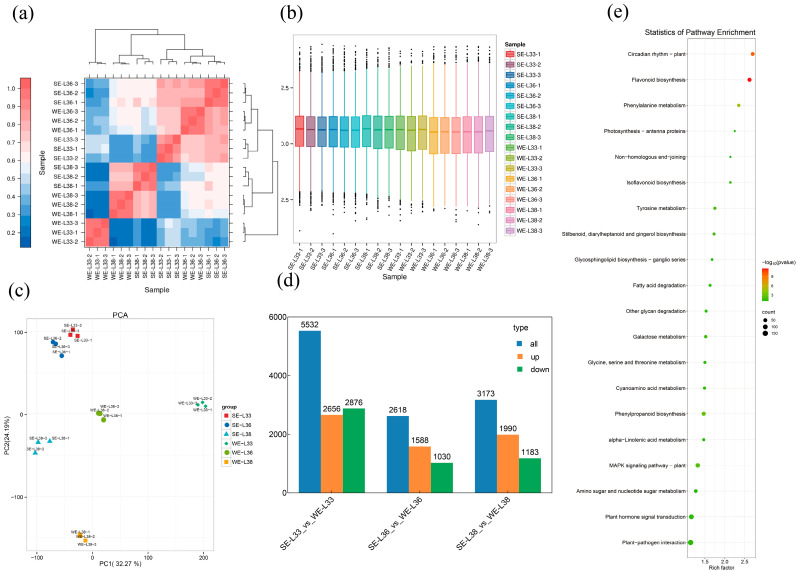
Analysis of gene expression profiles in the transcriptome. (**a**) Pearson correlation matrix of samples using transcriptome data. (**b**) Comparison analysis of gene expression levels across different experimental settings. (**c**) Principal component analysis (PCA) using gene expression levels. (**d**) Number of DEGs identified from seasonal comparisons. (**e**) Top 20 KEGG enrichment bubble map for DEGs between WE-L 38 and SE-L 38. Treatments: green berries from summer crop (SE-L33); full veraison in summer crop (SE-L36); harvest stage in summer crop (SE-L38); green berries from winter crop (WE-L33); full veraison in winter crop (WE-L36); and harvest stage in winter crop (WE-L38).

To obtain a more detailed understanding of the anthocyanin regulatory networks across the different growing seasons, the transcriptomes of the ‘Summer Black’ grapes were analyzed using RNA sequencing. RNA-Seq was performed, and a total of 120.82 GB of clean data was obtained. The GC content of the clean readings varied between 45.56% and 48.04%, while the proportion of Q30 bases exceeded 86.36% (Table S4). Upon aligning the valid data from all samples to the reference genome, we observed that the ratio of transcriptome data coverage to genome data coverage varied between 90.88% and 92.43% (Table S5). This indicates a high sequencing coverage and dependable data, which are acceptable for further experimental study. We evaluated the biological repeatability of the transcriptome data using the Pearson correlation coefficient (PCC). The findings indicated that the correlation coefficients between biological replicates within the same group were greater than 0.90 (Figure 3a, Table S6). This experiment revealed the variation in gene expression levels among several treatments. The results showed that the distribution of gene expression levels was relatively similar across the treatments, predominantly ranging from −2.5 to 4 (Figure 3b). Principal component analysis (PCA) of the identified genes (Figure 3c) exhibited strong consistency among the various treatment replicates. The treatments SE-L33, SE-L36, SE-L38, WE-L33, WE-L36, and WE-L38 were divided into distinct regions, suggesting notable variations among the treatments. The contribution values of the first principal component (PC1) and the second principal component (PC2) were 32.27% and 24.19%, respectively. On the PC1 axis, the distribution of WE-L33 and WE-L36 was positive, whereas the distribution of SE-L33, SE-L36, SE-L38, and WE-L38 was negative. On the PC2 axis, SE-L33, SE-L36, and WE-L33 showed a positive distribution, whereas SE-L38 and WE-L38 showed a negative distribution, indicating significant differences between the treatments.

### 2.4. KEGG Analysis Indicated That the Flavonoid Biosynthesis and Circadian Rhythm—Plant Pathways Are Significant Between the Summer and Winter Crops

Using the aforementioned transcriptome data, DEGs were identified by comparing gene expression levels between the summer and winter crops. DEGs were identified using a significance threshold of FDR < 0.05 and |log2fold change| > 1. A total of 11,323 DEGs were identified, which accounted for 35.64% of the annotated genes. In the E-L 33 stage, 2876 genes were suppressed and 2656 genes were activated in the winter crop berries compared to the summer crop berries. During the E-L 36 stage, there was an increase in the expression of 1588 genes and a decrease in the expression of 1030 genes in the winter crop berries compared to the summer crop berries. In the E-L 38 stage, there was an upregulation of 1990 genes and a downregulation of 1183 genes in the winter crop berries compared to the summer crop berries (Figure 3d). The KEGG enrichment pathways revealed that the DEGs in the comparison group of WE-L38 versus SE-L38 were mostly found in the following pathways: flavonoid biosynthesis, circadian rhythm—plant, phenylalanine metabolism, phenylpropanoid biosynthesis, and MAPK signaling pathway—plant (Figure 3e). The flavonoid biosynthesis and circadian rhythm—plant pathways were significant between the summer and winter crops.

### 2.5. Differentially Expressed Gene (DEG) Analysis in Circadian Rhythm—Plant Pathway and Anthocyanin Production Pathway in Double-Cropping Cultivation

**Figure 4:**
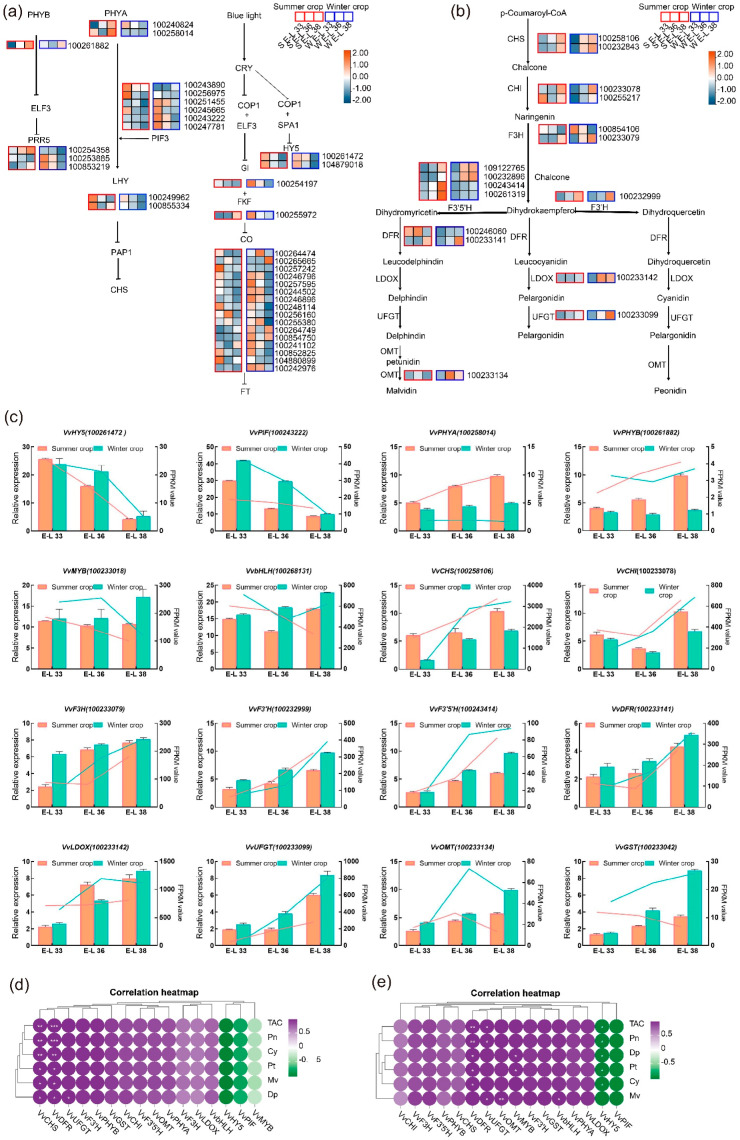
Differentially expressed genes (DEGs) in the circadian rhythm—plant pathway and flavonoid biosynthesis pathway under double-cropping cultivation. (**a**) DEGs in the circadian rhythm—plant pathway. (**b**) DEGs in the flavonoid biosynthesis pathway. Treatments: green berries from the summer crop (SE-L33) and full veraison in summer crop (SE-L36). (**c**) RT-qPCR validation of DEGs identified in RNA-seq analysis. (**d**) Pearson’s coefficient for summer crop. (**e**) Pearson’s coefficient for winter crop. Treatments: harvest stage in summer crop (SE-L38); green berries from winter crop (WE-L33); full veraison in winter crop (WE-L36); and harvest stage in winter crop (WE-L38). Orange boxes represent upregulated genes, while dark blue boxes represent downregulated genes. The red frame represents the summer crop, and the blue frame represents the winter crop. *, ** and *** indicate *p* ≤ 0.05, *p* ≤ 0.01, *p* ≤ 0.001, respectively.

In the circadian rhythm—plant pathway during the maturation process of the winter berries, the far-red light receptor phytochrome A (*VvPHYA*) and the circadian response gene pseudo-response regulator *VvPRR5* (100853219), along with the blue light receptor flavin-binding kelch repeat f-box1 (*VvFKF*), exhibited a declining pattern. Conversely, these receptors and genes displayed an increasing pattern during the maturation of the summer berries. Furthermore, genes associated with photoreceptors and the transmission of light signals, such as phytochrome interacting factor 3 (*VvPIF3*), circadian response gene (*VvLHY*), long hypocotyl 5 (*VvHY5*), circadian regulatory gene (*VvGI*), and circadian regulatory gene (*VvCO*), showed a declining pattern during the maturation of both the winter and summer berries. Red light receptor phytochrome B (*VvPHYB*) showed an increasing pattern during the maturation of both the winter and summer berries (Figure 4a and Table S7).

In the anthocyanin production pathway, *CHS*, *CHI* (100233078), *F3H* (100233079), *F3′H*, *F3′5′H*, *DFR* (100233141), *LDOX*, *UFGT*, and *OMT* showed an increasing pattern during the maturation of both the winter and summer berries (Figure 4b and Table S7).

Transcription factors (TFs) play a vital role in every phase of berry development. A total of 1915 TF genes were found to be differentially expressed across the different treatments. The TF genes were dispersed among 60 families of transcription factors. Through the examination of TF gene expression levels, five TF families with the most significant differential expression were observed. These families were MYB (with 283 members), MYB-related (with 232 members), AP2-EREBP (with 148 members), bHLH (with 133 members), and NAC (with 85 members) (Table S8).

### 2.6. Strong Consistency Between the Expression Levels Obtained from RT-qPCR and Those Obtained from the Transcriptome Analysis

In order to confirm the accuracy of the transcriptome data, a total of 16 differentially expressed genes (DEGs) were chosen for validation using RT-qPCR. The expression levels of four genes in the circadian rhythm—plant pathway were examined in the summer and winter crops (Figure 4c). The expression levels of *VvHY5* and *VvPIF* were detected to be higher in winter compared to summer; in the E-L36 stage, *VvHY5* was 1.32 times greater in winter than in summer. *VvHY5* and *VvPIF* declined continuously from the beginning of veraison to the harvest stage in both the summer crop and winter crop. In contrast, the relative expression of *VvPHYA* and *VvPHYB* exhibited higher expression levels in summer compared to winter, consistently increasing from the beginning of veraison to the harvest stage throughout summer. Meanwhile, *VvMYB* and *VvbHLH* showed a higher expression in winter compared to summer. In the E-L36 stage, the expressions of *VvMYB* and *VvbHLH* were 1.17 and 1.34 times greater in winter than in summer, respectively, with a continuous increase in the relative expression from the beginning of veraison until the harvest stage.

The expression levels of 10 genes in the anthocyanin biosynthesis pathway were assessed in both the summer and winter crops (Figure 4c). The expression levels of *VvF3H*, *VvF3′H*, *VvF3′5′H*, *VvDFR*, *VvLDOX*, *VvUFGT*, *VvOMT*, and *VvGST* were greater in the winter crop compared to the summer crop. In the E-L36 stage, the expressions of *VvDFR* and VvOMT were 1.34 and 1.28 times greater in winter than in summer, respectively, and *VvUFGT* showed the highest expression in winter, which was 1.98 times greater than that in summer. Conversely, *VvCHS* and *VvCHI* exhibited higher expression levels in the summer crop compared to the winter crop. The relative expressions of *VvCHS*, *VvF3H*, *VvF3′H*, *VvF3′5′H*, *VvDFR*, *VvLDOX*, *VvUFGT*, *VvGST*, and *VvOMT* consistently rose from the beginning of veraison until the harvest stage in both seasons. *VvCHI* exhibited a decrease and then an upswing from the full veraison to the harvest phase, in both summer and winter. The results show strong consistency between the expression levels obtained from RT-qPCR and those obtained from the transcriptome analysis.

### 2.7. Examining the Correlation Between the Total or Individual Levels of Anthocyanins and Genes Involved in the Anthocyanin Biosynthetic Pathway

The Pearson correlation coefficient was calculated to analyze the associations between different variables (Figure 4d). In the summer crop, positive associations were found between anthocyanin content and the *VvCHS*, *VvDFR*, and *VvUFGT* genes. A strong positive relationship was observed between TAC, Pn-3-G content, and the *VvDFR* gene in the summer crop (*p* < 0.001). Significant positive relationships were identified between Dp-3-G content and the *VvUFGT* gene (*p* < 0.05).

In the winter crop, significant positive correlations were found between anthocyanin content and the genes *VvDFR*, *VvUFGT*, *VvOMT*, *VvMYB*, and *VvbHLH*, and significant negative correlations were noted between TAC, Pn-3-G, Dp-3-G, Pt-3-G, and Cy-3-G contents, and the *VvHY5* gene. A strong positive correlation was detected between Mv-3-G content and the *VvOMT* gene (*p* < 0.01). Furthermore, significant positive correlations were noted between Mv-3-G content and the expression of the *VvUFGT* and *VvbHLH* genes (*p* < 0.05) in the winter crop (Figure 4e).

### 2.8. Seasonal Treatments and Pruning Techniques Improve Grape Quality and Gene Expression Involved in Anthocyanin Biosynthetic Pathways in Double-Cropping Cultivation

**Figure 5:**
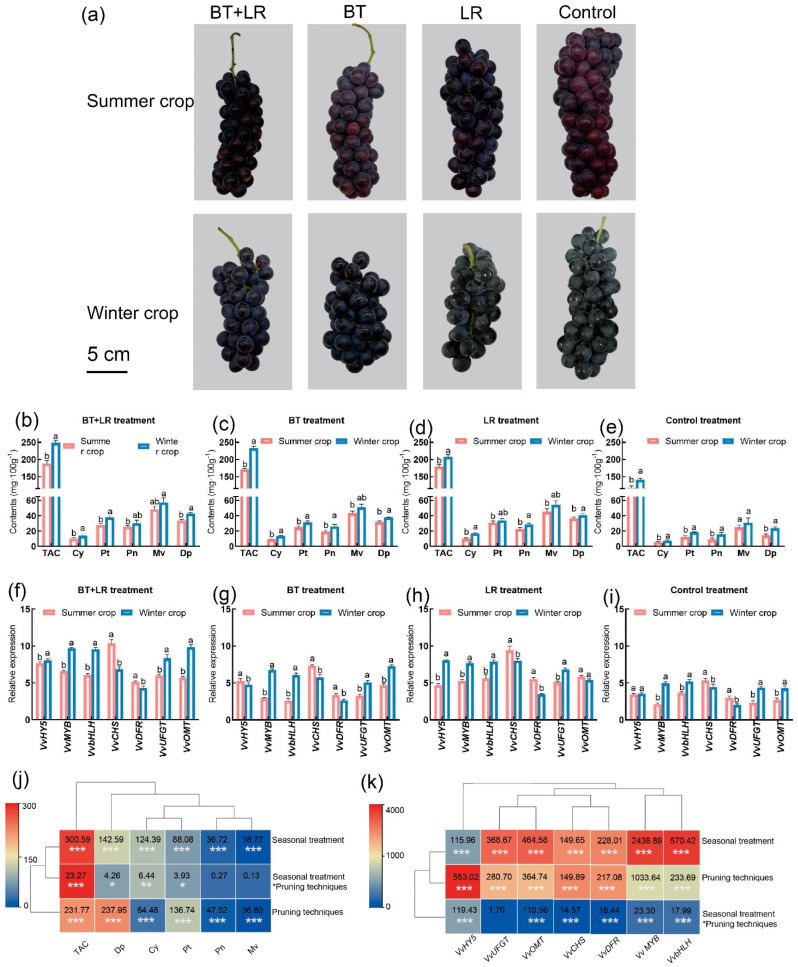
The effect of berry thinning (BT), basal leaf removal (LR), and their combination on the content of anthocyanins in ‘Summer Black’ grape berries in the harvest stage (E-L 38). (**a**) Visual appearance of representative bunches of ‘Summer Black’ table grapes harvested from vines subjected to treatments of berry thinning (BT), basal leaf removal (LR), and their combination. (**b**) BT+LR treatment anthocyanin content. (**c**) BT treatment anthocyanin content. (**d**) LR treatment anthocyanin content. (**e**) Control treatment anthocyanin content. (**f**) BT+LR treatment anthocyanin-related gene expression. (**g**) BT treatment anthocyanin-related gene expression. (**h**) LR treatment anthocyanin-related gene expression. (**i**) Control treatment anthocyanin-related gene expression. (**j**) Test of intersubjective effects of seasonal treatments and pruning techniques on anthocyanin content. (**k**) Test of intersubjective effects of seasonal treatments and pruning techniques on related gene expression. Developmental stages: green berries (E-L 33); full veraison (E-L 36); and harvest stage (E-L 38). The vertical bar denotes the standard error of the mean (SEM). Statistical significance is denoted by different letters (*p* < 0.05). *, ** and *** indicate *p* ≤ 0.05, *p* ≤ 0.01, *p* ≤ 0.001, respectively.

Under the BT and LR treatments, the summer and winter berries differed significantly in appearance and color, with a deeper skin color in the winter crop and a larger size and weight in the summer crop (Figure 5a). The winter crop berries showed elevated levels of TSS and pH compared to the summer berries. The summer crops had larger yields per vine, individual berry weights, berry diameters, and berry lengths than the winter crops (Table S9). In the harvest stage, the winter crops exhibited the highest total anthocyanin content. Specific monomeric anthocyanins, such as Cy-3-G, Pn-3-G, Mv-3-G, Dp-3-G, and Pt-3-G, were significantly higher in the winter crop berries (Figure 5b–e). The genes *VvMYB*, *VvbHLH*, *VvUFGT*, and *VvOMT* exhibited increased expression levels in the winter crop under the BT+LR, BT, LR, and control treatments. *VvCHS* and *VvDFR* exhibited decreased expression levels in the winter crop under the BT+LR, BT, LR, and control treatments (Figure 5f–i).

The individual berry weights of the BT and BT+LR treatments were lower compared to those of the LR and control treatments (Figure 5a, Table S9). Both BT and LR increased the TSS content of the berries. The BT and LR treatments reduced grape yield per vine while increasing the size and weight of individual berries. Additionally, the BT and LR treatments reduced grape acidity (Table S9). The BT+LR treatment had the most substantial influence on anthocyanin levels and their individual compounds, followed by the BT and LR treatments individually (Figure 5b–e); the control treatment had the lowest content. Vines treated with the combination of berry thinning (BT) and basal leaf removal (LR) exhibited the highest gene expression of *VvMYB*, *VvbHLH*, *VvUFGT*, and *VvOMT*, followed by vines treated with LR and BT alone. The control vines had the lowest expressions of *VvMYB*, *VvbHLH*, *VvUFGT*, and *VvOMT* (Figure 5f–i).

Figure 5j,k show that the differences in the total anthocyanin content and expression of the *VvMYB*, *VvbHLH*, *VvDFR*, *VvUFGT*, and *VvOMT* genes between the seasonal treatments are more significant (*p* < 0.01) than those between the pruning treatments when comparing the F scores (Figure 5j,k, Table S10).

## 3. Discussion

### 3.1. Grape Berries from the Winter Crop Accumulated More Anthocyanins than Those from the Summer Crop Due to the Lower Temperature

Appropriate low temperatures can promote anthocyanin accumulation in plants. Our results showed that winter temperatures are lower than summer temperatures during the harvesting period. In the summer season, increased yields and berry sizes were observed; the primary reasons are that the fruit expansion period in summer is longer than in winter, and the temperatures during the summer fruit expansion period are more suitable than those in winter. Meanwhile, the berries harvested in winter had a higher total anthocyanin content, as well as higher levels of individual anthocyanin components. These results are supported by previous research which found that mild temperatures around 15 °C [29,30] enhance anthocyanin accumulation, whereas high temperatures usually exacerbate plant catabolism and inhibit anthocyanin synthesis [31,32,33,34,35]. Elevated greenhouse temperatures in summer can increase water loss in vine plants and reduce the production of aromatic compounds and anthocyanins in berries, leading to an inferior taste in table grapes [36].

Temperature directly regulates anthocyanin synthesis by regulating structural genes and enhancing gene expression during winter. Our study revealed that the winter crop was exposed to lower temperatures compared to the summer crop, particularly from the green berry stage to the grape harvest. Anthocyanin biosynthetic pathway genes, such as *VvF3H*, *VvF3′H*, *VvF3′5′H*, *VvDFR*, *VvLDOX*, *VvUFGT*, *VvOMT*, and *VvGST*, exhibited higher expression levels in winter. In the winter berries, the higher expression of *VvUFGT* led to an increased content of Cy-3-G, and the higher expression of the *VvOMT* gene led to an increased content of Mv-3-G. A low temperature of 15 °C enhanced the gene expression of *VviF3′H*, *VviF3′5′H2*, *VviUFGT*, and *VviFAOMT* in *Vitis vinifera* [37]. A low temperature of 15 °C significantly increased the expression of the *BsPAL*, *BsCHS*, *BsF3H*, and *BsANS* genes in Begonia semperflorens, and the upregulation of these genes led to anthocyanin accumulation [38]. The *UFGT* activity of ‘Fuji’ apples was enhanced under treatment with a low temperature of 15 °C at night [39]. A high temperature of 32 °C inhibited the transcription of *OsCHS*, *OSF3′H*, *OsDFR*, and *OsANS* in rice grains and reduced the cyanin content in rice grains [40]. The expression of structural genes such as *CHS*, *CHI*, *DFR*, and *LDOX* was generally inhibited under a high-temperature treatment of 28 °C. The decrease in *LDOX* transcription led to a decrease in anthocyanin accumulation [41]. Under treatment with a high temperature of 35 °C, *VviUFGT* gene expression decreased, enzyme activity decreased, and anthocyanin content decreased [37]. At a high temperature of 30 °C, the expression of *CyDFR*, *CyANS*, *CyMYB1*, and *CybHLH2* in *Cymbidium* was inhibited, resulting in a decrease in anthocyanin accumulation in the perianth [42]. The results showed that there were different expression patterns of structural genes under different temperatures.

**Figure 6:**
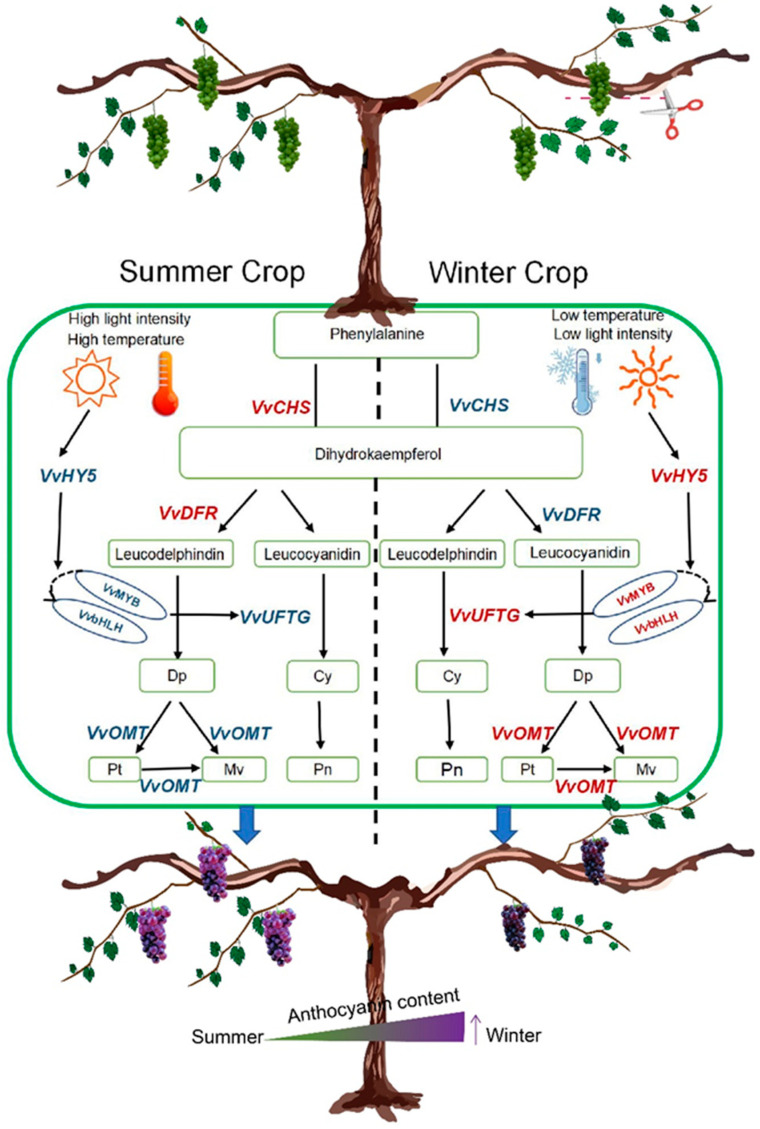
The molecular mechanism of the regulation of grape anthocyanin metabolism: cyanidin-3-O-glucoside (Cy-3-G), peonidin-3-O-glucoside (Pn-3-G), malvidin-3-O-glucoside (Mv-3-G), delphinidin-3-O-glucoside (Dp-3-G), and petunidin-3-O-glucoside (Pt-3-G).

Temperature indirectly regulates anthocyanin synthesis by regulating transcription factors and enhancing *VvHY5*, *VvMYB*, and *VvbHLH* gene expression during winter. Our study revealed that *VvHY5* and *VvPIF* showed higher expression levels in the winter crop compared to the summer crop, with *VvMYB* and *VvbHLH* transcription factors also maintaining elevated expression levels in winter. High temperature can inhibit anthocyanin biosynthesis by disrupting the stability of the HY5 protein [41]. HY5 also interacts with phytochrome interacting factor 3 (*PIF3*) to co-regulate downstream target genes in the anthocyanin biosynthetic pathway, thereby promoting anthocyanin synthesis [43]. Transcription factors such as *HY5* are also involved in the regulation of anthocyanin synthesis; under UV-B irradiation, *ZbHY5* could regulate the expression levels of structural genes involved in anthocyanin biosynthesis by combining with *ZbMYB113*, thereby affecting anthocyanin accumulation [44]. *HY5* binds to the promoters of transcription factors such as *PAP1*, *MYBL2*, and *MYB10* and affects their expression [45,46,47]. There is an interaction between *CyMYB1* and *CybHLH2* [42]. The N-terminal of the *bHLHⅢf* subgroup binds to the R3 region in R2R3-MYB, working with *MYB* [48]. In peaches (*Prunus persica*), *PpMYB6*, *PpMYB44*-like, *PpbHLH35*, *PpbHLH36*-like, and *PpbHLH51* work together to activate the *PpUFGT* promoter and induce anthocyanin accumulation [49] (Figure 6).

### 3.2. Berry Thinning and Basal Leaf Removal Improve Anthocyanin Content by Enhancing Expression Levels of Genes Involved in Anthocyanin Production

Basal leaf removal targets the lower leaves, whereas the BT technique involves selectively removing the upper parts of the clusters. Our results indicate that the chemical composition of grapes from the BT+LR, LR and BT treatments had significantly higher levels of anthocyanins and TSS compared to the control group, while the acidity of berries in the treated vines was lower than that of the control, consistent with prior research finding that berry thinning and basal leaf removal can improve grape flavor [50,51,52]. Proanthocyanidin concentrations tended to increase with an increase in berry thinning [52]. Positive effects of berry thinning on advanced fruit maturity, decreased acidity, and increased anthocyanin and phenolic compound contents in Cabernet Sauvignon have been reported [53]. BT practices can directly influence the vine’s source/sink ratio, potentially enhancing photosynthetic uptake and allocation, where the vines could produce and allocate sufficient anthocyanin levels to each berry. Leaf removal is a modern centralized vineyard management technology. Its main purpose is to improve porosity in dense canopies, to allow light exposure [50] and air circulation, and to enhance berry color formation, as well as berry ripening [54]. Some older leaves stop providing photosynthetic products to the fruit during the coloring phase of grape development, and too many leaves can block a large amount of sunlight from reaching the fruit. Thus, increasing the amount of light exposure to the fruit and making it more uniform can be achieved by trimming off any excess old leaves or those that obstruct light absorption.

Our results indicate that the expression levels of *VvMYB*, *VvbHLH*, *VvUFGT*, and *VvOMT* in the BT and LR treatments were significantly higher than those in the control, both individually and in combination. Previous research demonstrated that cluster thinning resulted in higher anthocyanin accumulation, with the *F3′H*, *F3′5′H*, *UFGT*, and *OMT* genes showing significant upregulation in treated grapevines compared to untreated controls [7]. The litchi *LcbHLH1* and *LcbHLH3* transcription factors and *LcMYB1* work together to promote anthocyanin accumulation [55]. The gentian *GtbHLH1* protein can interact with *GtMYB3* to promote anthocyanin synthesis in gentian petals [56]. The *MYB* and *bHLH* transcription factors modulate berry pigmentation by controlling the expression of *UFGT* [57]. Our findings underscore the critical roles of these genes in anthocyanin production and highlight the significant effects of pruning techniques on gene expression and anthocyanin accumulation in grape berries (Figure 6).

## 4. Materials and Methods

### 4.1. Experimental Site and Plant Material Under Double-Cropping Cultivation

The experiment was conducted in a vineyard of Lunong Agricultural Technology Co., Ltd., in Xiamen, Fujian, China, which is located at 24°51′ N, 117°59′ E. The climate in this area is humid subtropical monsoon. The orchard grows ‘Summer Black’ table grapes. The grapes are planted under greenhouse protection cultivation. The vines were trained on a T-shaped horizontal trellis system.

The double-cropping cultivation method for grapes is a scientifically managed and optimized planting technique that uses grape winter buds to induce flowering, producing two harvests within a year. Under greenhouse protection cultivation in southern China, the management of summer berries includes medium and short pruning in January, bud forcing in February, flowering and fruit setting from April to June, and harvesting of summer berries from June to July. After harvesting the summer berries, fertilization is applied to restore the vigor of the vines. In July, reasonable long branch pruning and cyanamide treatments are carried out to promote the rapid sprouting of winter buds, entering the second growth cycle. The winter berries mature in December, achieving the second harvest [28]. The vines were managed using regular agricultural practices such as fertilization, irrigation, soil management, pruning, and disease control, which remained consistent throughout the summer and winter seasons.

At the front, middle, and back of the experimental greenhouses, temperature sensors (Shandong Zhongtian Internet of Things Co. Ltd., Weihai, China) were installed. The sensors were placed between the vines and the canopy steel frame. The temperature was measured at 8 h intervals.

### 4.2. Grape Berry Sampling Stages

Grape berry samples were collected from the summer and winter crops at five different development stages as follows: (A) green berries (E-L 33); (B) the beginning of veraison (E-L 35), characterized by the softening and change in the color of the berries; (C) full veraison (E-L 36); (D) the end of veraison (E-L 37), characterized by the presence of soft and fully colored berries; and (E) the harvest stage (E-L 38) [58,59] at the time of harvest. Each stage involved collecting 15 grape clusters. After the harvest, the berry samples were placed in chilled containers and promptly transported to the laboratory for analysis.

### 4.3. Experimental Design and Treatments of Pruning Techniques

This study employed a randomized block design comprising three replicates of four treatments, with nine vines in each treatment. All treatments, such as reducing the number of berries and removing the basal leaves, were carried out during the pea stage (E-L 31) of the plants. The treatments applied were as follows:

BT: Consisted of cutting the top third of each cluster on the vine.

LR: Consisted of removing the initial five basal leaves from every shoot.

BT+LR: Involved cutting the top of each cluster on the vine and then removing the initial five basal leaves from every shoot.

Control: Consisted of vines that received no treatment.

Grape berry samples were collected from the summer and winter crops in the harvest stage (E-L 38). Each treatment involved collecting 15 clusters. After the harvest, the berry samples were placed in chilled containers and promptly transported to the laboratory for analysis.

### 4.4. Determination of Berry Quality Characteristics

The yield per vine was calculated using the average weight of the clusters and the number of clusters. The individual berry weight was determined by dividing the per vine weight by the number of berries per vine. For each treatment, a total of 60 berries were used and divided into three biological replicates to measure berry diameter and berry length. The remaining berries were manually crushed to extract juice for analyzing their chemical characteristics, including TSS, pH, and titratable acidity. TSS was measured using a portable refractometer (DR301-95, Krüss Optronic, Hamburg, Germany), and the results were reported in Brix units. The juice pH was determined using a pH meter. Total acidity was assessed by visual titration with 0.1 mol/L NaOH until the pH reading on the meter reached 8.2. The acidity values were then expressed as a percentage relative to tartaric acid.

### 4.5. Preparation of Skin Samples for Analysis of Total Anthocyanins and Individual Anthocyanins

The berries were de-skinned using tweezers and a knife and then cryopreserved in liquid nitrogen for testing the anthocyanin content. The total anthocyanin content was measured using the pH differential method [60]. The mono-glucosides of the five main individual anthocyanins, namely cyanidin-3-O-glucoside (Cy-3-G), peonidin-3-O-glucoside (Pn-3-G), malvidin-3-O-glucoside (Mv-3-G), delphinidin-3-O-glucoside (Dp-3-G), and petunidin-3-O-glucoside (Pt-3-G), were quantified using a high-performance liquid chromatography (HPLC) system (LC-100; Wufeng series, Shanghai, China). The HPLC system was equipped with an LC-P100 pump and operated by Empower 3 software.

HPLC was conducted as previously described [60]. Briefly, a 20 µL aliquot of grape skin extract was dissolved in methanol, acidified with 1% hydrochloric acid, and then fed into the HPLC system. The samples were subsequently isolated using a Brisa LC2 C18 column (250 × 4.6 mm, 5 µm particle size) utilizing a gradient of two eluents, A and B. Eluent A consisted of acetonitrile/water/formic acid in a volumetric ratio of 50:45:5, while eluent B consisted of water/formic acid in a volumetric ratio of 95:5. The gradient conditions were as follows: The concentration of A was 0–3% at 1 min, 3–15% at 11 min, 15–25% at 12 min, 25–30% at 4 min, and 30% at 7 min. The concentration remained for 5 min before returning to the initial circumstances. The volumetric flow rate was 1.0 milliliters per minute at a temperature of 30 °C, and the wavelength used for detection was 520 nm. The identification of peaks was accomplished by comparing the UV–Vis spectrum peaks of the samples with those of standard spectra and aligning their retention durations. The quantification of anthocyanin content was determined by measuring the peak regions of external standards. The peak area was plotted against concentration to generate linear calibration curves. The ultimate anthocyanin concentration was quantified as milligram per 100 g of fresh weight.

### 4.6. Transcriptome Analysis of Grape Berries in Different Growth Stages Under Double-Cropping Cultivation

Grape berries were collected in the green berry stage (E-L 33), full veraison stage (E-L 36), and harvest stage (E-L 38) from the summer and winter crops. The berries were de-skinned using tweezers and a knife and then cryopreserved in liquid nitrogen for RNA-seq analysis. The analysis was conducted by Beijing Biomarker Technologies Corporation accessed on 17 June 2024 (www.biomarker.com.cn, Beijing, China). A sample weighing 0.3 g was pulverized using liquid nitrogen, and the total RNA was isolated using the RNA prep Pure Plant Kit (Tiangen, Beijing, China). The RNA’s purity and concentration were evaluated using a NanoDrop 2000 spectrophotometer (Thermo Scientific, WLM, USA). The RNA integrity was assessed using the Tanon 2500 gel imaging system (Tanon, Shanghai, China). The library was constructed using the TruSeq Stranded mRNA LT Sample Prep Kit (Illumina, S.F., USA) following the instructions provided by the manufacturer. The reference genome utilized in this study was the publicly accessible version of the full *V. vinifera* genome at 12× coverage, provided by the French–Italian Public Consortium for Grapevine Genome Characterization (ftp://ftp.ensemblgenomes.org/pub/release-23/plants/fasta/vitis_vinifera/dna/, accessed on 1 June 2024). The DESeq2 program was utilized to perform differential expression analysis between the control and treatment groups. Differentially expressed genes (DEGs) were identified using a significance threshold of FDR < 0.05 and |log2fold change| > 1. The GO seq R package and KOBAS 3.0 software were utilized to conduct enrichment analyses for gene ontology (GO) and Kyoto Encyclopedia of Genes and Genomes (KEGG), respectively.

### 4.7. Quantitative Real-Time PCR Analysis

Primer 5.0 was used to design specific primers for RT-qPCR analysis. The primer pairs and corresponding amplicon lengths for the target genes are listed in Table S1. RT-qPCR analysis was performed using an ABI 7500 Real-time Detection System (Thermo Fisher Scientific, USA). A total of 1 μg of total RNA was used as a template, and the Takara Prime Script™ RT Reagent Kit with gDNA Eraser (Takara in Dalian, China) was used to synthesize cDNA according to the manufacturer’s instructions. Takara SYBR Premix Ex TaqTM II (Takara in Dalian, China) and ROX Plus (Takara in Dalian, China) were used as qPCR reaction reagents. Each reaction mixture consisted of 0.5 μL of each forward and reverse primer, 2 μL of diluted cDNA, 10 μL of SYBR Premix Ex Taq™ II, and 7.0 μL of sterile distilled H_2_O, giving a total of 20 μL. PCR was performed using thermal cycling parameters of 95 °C for 30 s, followed by 45 cycles of 95 °C for 5 s and 60 °C for 40 s. To confirm the specificity, a melting curve was generated by gradually increasing the temperature from 60 to 99 °C in 0.5 °C increments. This was carried out at the end of the reaction and held for 1 s to ensure that the product was evenly distributed. Each RT-qPCR sample was analyzed using a total of nine duplicates, consisting of three biological and three technical replicates. The 18S rRNA gene served as an internal reference. The 2^−∆∆CT^ method was used to assess the relative quantification of target gene expression. Gene expression levels are presented as means with standard errors, based on three replicates.

### 4.8. Statiistical Analyses

Statistical analyses for this investigation were conducted using SPSS 19.0 (SPSS Inc., Chicago, IL, USA). A model graph was created using Adobe Illustrator Artwork 25.0.

## 5. Conclusions

Grapes from the winter crop accumulated higher anthocyanin contents than those from the summer crop. This was a result of the lower temperatures in winter, which can activate a greater number of genes associated with anthocyanin synthesis and promote the production of their monomers. The differences in the total anthocyanin content and gene expression between the seasonal treatments were more significant than those between the pruning treatments.

## Data Availability

Data will be made available on request.

## Supplementary Materials

The following supporting information can be downloaded at: www.mdpi.com/article/10.3390/plants14010026/s1, Lists the primers used for quantifying gene expression levels through RT-qPCR (Table S1), Displays the mean daily temperature throughout the growth of grape berries (Table S2), Berry quality characteristics and berry chemical composition in winter and summer cropping cycle(Table S3), All Sample_GC_Q.stat (Table S4), Statistical table of sequence comparison results between sample sequencing data and selected reference genomes (Table S5), Sample correlation (Table S6), Differentially expressed genes (DEGs) in the circadian rhythm—plant pathway and flavonoid biosynthesis pathway in double-cropping Cultivation (Table S7), Transcription factors (TFs) statistics (Table S8), Effect of berry thinning and basal leaf removal on vegetative-reproductive indices and berry chemical composition in winter and summer cropping cycle (Table S9), Test of intersubjective effects (Table S10) (XLSX).

## Abbreviations

BT, basal berry thinning; *CHS*, chalcone synthases; *CHI*, chalcone isomerases; *CO*, zinc finger protein constans-like; Cy-3-G, cyanidin-3-O-glucoside; DEGs, differentially expressed genes; *DFR*, dihydro flavonol reductase; Dp-3-G, delphinidin-3-O-glucoside; *F3H*, flavanone-3-hydroxylase; *F3′H*, flavonoid 3′-hydroxylase; *F3′5′H*, flavonoid 3′5′-hydroxylase; *FKF*, flavin-binding kelch repeat f-box1; GI, protein gigantea; *GST*, glutathione S-transferase; HPLC, high-performance liquid chromatography; *HY5*, long hypocotyl 5; *LDOX*, leucoanthocyanidin dioxygenase; *LHY*, late elongated hypocotyl; LR, leaf removal; Mv-3-G, malvidin-3-O-glucoside; *OMT*, O-methyltransferase; Pt-3-G, petunidin-3-O-glucoside; PCA, principal component analysis; PC1, first principal component; PC2, second principal component; *PHYA*, phytochrome A; *PHYB*, phytochrome B; *PIF3*, phytochrome interacting factor 3; Pn-3-G, peonidin-3-O-glucoside; *PRR5*, pseudo-response regulator; RT-qPCR, quantitative real-time PCR; TAC, total anthocyanins; TFs, transcription factors; TSS, total soluble solids; *UFGT*, UDP-glucose flavonoid 3-O-glucosyltransferase.
